# Retraction: Human and Environmental Dangers Posed by Ongoing Global Tropospheric Aerosolized Particulates for Weather Modification

**DOI:** 10.3389/fpubh.2016.00156

**Published:** 2016-07-20

**Authors:** Frontiers Editorial Office

The journal retracts the 30 June 2016 article cited above. Based on information discovered after publication and reported to Frontiers in July 2016, the article was examined, revealing that the complaints were valid and that the article does not meet the standards of editorial and scientific soundness for Frontiers in Public Health. The retraction of the article was approved by the Field Chief Editor of Frontiers in Public Health and the Specialty Chief Editor of Environmental Health. The author considers the retraction to be unwarranted and therefore does not agree to the statement.

